# The privileged immunity of immune privileged organs: the case of the eye

**DOI:** 10.3389/fimmu.2012.00296

**Published:** 2012-09-21

**Authors:** Inbal Benhar, Anat London, Michal Schwartz

**Affiliations:** Department of Neurobiology, Weizmann Institute of ScienceRehovot, Israel

**Keywords:** immune privilege, visual system, immunomodulation, neuroprotection and neuronal repair, inflammation

## Abstract

Understanding of ocular diseases and the search for their cure have been based on the common assumption that the eye is an immune privileged site, and the consequent conclusion that entry of immune cells to this organ is forbidden. Accordingly, it was assumed that when immune cell entry does occur, this reflects an undesired outcome of breached barriers. However, studies spanning more than a decade have demonstrated that acute insults to the retina, or chronic conditions resulting in retinal ganglion cell loss, such as in glaucoma, result in an inferior outcome in immunocompromised mice; likewise, steroidal treatment was found to be detrimental under these conditions. Moreover, even conditions that are associated with inflammation, such as age-related macular degeneration, are not currently believed to require immune suppression for treatment, but rather, are thought to benefit from immune modulation. Here, we propose that the immune privilege of the eye is its ability to enable, upon need, the entry of selected immune cells for its repair and healing, rather than to altogether prevent immune cell entry. The implications for acute and chronic degenerative diseases, as well as for infection and inflammatory diseases, are discussed.

## INTRODUCTION

Over the past decades, the mammalian central nervous system (CNS), including the eye, brain, and spinal cord, were believed to be sealed from the circulation. Thus, immune activity at these sites was considered forbidden, and was collectively assumed to be consistently detrimental. As a consequence, the inflammatory response in the eye or the brain was assessed solely based on counting the number of immune cells, without regard to their phenotype or function. Thus, the poor ability of the optic nerve to regenerate following injury, as well as the poor recovery following acute injury to any other parts of the CNS, were assumed to be an outcome of local detrimental immune activity seen at the lesion site (Fitch et al., 1999; Popovich et al., 1999; Ghirnikar et al., 2001). Such a view was almost universally accepted from the early 1980s and supported the use of anti-inflammatory drugs to treat victims of CNS injuries (Constantini and Young, 1994; Carlson et al., 1998).

With time and the advance of technologies, there was an increase in the understanding of the heterogeneity of innate and adaptive immunity in general, and in the CNS in particular, with respect to both functional cell subsets (Korn et al., 2007; Gee et al., 2008; O’Shea et al., 2008; Auffray et al., 2009; Prinz et al., 2011; Zhu et al., 2011) and origin (Geissmann et al., 2010; Ginhoux et al., 2010; Prinz et al., 2011). As a corollary, it became clear that some of the blanket assumptions regarding the eye and the brain were not accurate, and, accordingly, that some experimental findings had not been properly interpreted. Thus, it became evident that the response to CNS injury, similar to that in other tissues in the body, is a multi-step process that requires a set sequence, and synchrony of events in time and space; many of the steps that take place in the healing process following “sterile” injuries are similar if not identical to processes occurring outside the CNS with respect to the immune response (Dusart and Schwab, 1994; Frank and Wolburg, 1996; Arnold et al., 2007; Nahrendorf et al., 2007; Rolls et al., 2009; Shechter et al., 2009; Stirling et al., 2009; London et al., 2011). The early innate immune response involves cells that are needed for cleaning the lesion site, yet the activity of these cells must be followed by immune cells that terminate this initial response and subsequently contribute to the repair. Both stages involve innate immune cells of distinct phenotypes; the cells that contribute to the termination of the local early response are largely monocyte-derived macrophages that acquire and exert a local anti-inflammatory function (Kigerl et al., 2009; Shechter et al., 2009; London et al., 2011; Zhu et al., 2011). The obvious question is how such a response can be reconciled with the traditional view of the eye as an immune privileged site; do these findings change our understanding of the privilege, or do they require breaking of privilege under severe conditions? Here, focusing on the eye, we will discuss a different view of the physiological meaning of the CNS as an immune privileged site, and its manifestations under pathological conditions.

## THE EYE AS AN IMMUNE PRIVILEGED ORGAN

Immune privileged organs were operationally defined as sites in the body where foreign tissue grafts can survive for extended, often indefinite periods of time, whereas similar grafts placed at regular sites in the body are acutely rejected (Medawar, 1948). These organs include the eye and the brain, as well as the pregnant uterus, testis, and several others (Streilein, 2003b; Niederkorn, 2006). Such immune privilege is thought to be an evolutionary adaptation to protect tissues that are indispensable, yet have limited regeneration capacity, like the brain and the eye, from the potentially damaging effects of an uncontrolled inflammatory immune response. Thus, immune privileged organs were considered as ones to which immune cell entry is forbidden; leukocytes were believed to be excluded from these vital organs by the presence of specialized physical barriers, the blood–tissue barriers.

In contrast to the previous view that immune privilege is maintained by immune cell exclusion, it is now increasingly accepted that the privileged status is preserved by local active mechanisms that suppress responses to antigens within the privileged tissues (Niederkorn and Stein-Streilein, 2010). In the eye, one such mechanism is anterior chamber-associated immune deviation (ACAID), referring to a phenomenon in which antigenic material introduced into the anterior chamber of the eye elicits a systemic immune response that results in immune deviation, characterized by the suppression of T cell-mediated immunity, while enabling the production of non-complement-fixing antibodies (Kaplan et al., 1975; Streilein, 2003b; Niederkorn, 2006). ACAID involves the migration of specialized antigen presenting cells from the eye to the thymus and spleen, and is associated with an elevation in regulatory, γδ, and natural killer T cells (Streilein, 2003b; Niederkorn, 2006). Other mechanisms aimed at maintaining the immune privileged state of the eye include the reduced expression of MHC molecules on ocular cells, and the existence of an intraocular anti-inflammatory environment, mediated by resident cells, and various molecules, both surface-bound and soluble, all of which serve to modulate the activity of infiltrating immune cells, *in situ* (Streilein, 2003b; Schewitz-Bowers et al., 2010; Zhou et al., 2012). These well-orchestrated, multifaceted mechanisms, known to involve numerous pathways, were long thought to be designed to ensure limited infiltration of circulating immune cells to the eye, leaving behind a tissue that was considered autonomous in terms of repair. It is puzzling, however, why a fragile and precious organ such as the eye would evolve such complex tolerance mechanisms, if their sole purpose were to guarantee immune ignorance. Moreover, several studies have shown that immunocompromised mice exhibit worse recovery from optic nerve and retinal insult than do their immunocompetent counterparts (Kipnis et al., 2001; Schori et al., 2001; Yoles et al., 2001), similar to the case in peripheral nerve injury (Serpe et al., 1999). Similarly, recent studies have demonstrated that well-regulated immune responses in the CNS, rather than immune ignorance, are optimal for the recovery of the tissue after insult, whether sterile or immune-induced (Kerr et al., 2008; Shechter et al., 2009; Caspi et al., 2011; London et al., 2011; London et al., under revision). Thus, it is becoming increasingly clear that immune privilege is not aimed at entirely suppressing immune responses in the target organ, but rather at maintaining a specialized, tightly regulated immunological niche to preserve the integrity of especially vulnerable organs, such as the brain and the eye (Streilein, 2003b; Niederkorn, 2006).

## REGULATED IMMUNE RESPONSES ARE BENEFICIAL IN MITIGATING EYE PATHOLOGIES

Inflammation is the body’s adaptive response to any insult, be it mechanical, biochemical, or immune-mediated. However, inflammation is beneficial only on the condition that it ends in active resolution (Gronert, 2010). Studies on wound healing outside the CNS have characterized distinct subsets of macrophages that infiltrate the site of injury and display different functions corresponding to the changing needs of the tissue along the course of healing; these include the clearing of dead cells and tissue debris at the first stage, and the secretion of anti-inflammatory cytokines and growth factors at the later stage, to aid tissue regrowth and restoration of immune homeostasis (Arnold et al., 2007; Nahrendorf et al., 2007). Recently, our team demonstrated that a subset of monocyte-derived macrophages, which manifests an immune-resolving phenotype, is essential for the resolution of inflammation after sterile insults, in models of spinal cord injury and retinal glutamate intoxication (Shechter et al., 2009; London et al., 2011). In both of these cases, such macrophages were found to be crucial for recovery, as was measured by a functional motor scale after spinal cord injury, and directly in terms of cell survival in the retina. Thus, despite the classification of these organs as immune privileged, they nevertheless derive benefit from the controlled recruitment of innate immune cells from the circulation, to assist in their healing. Notably, while the CNS contains its own population of immune cells, the resident microglia, we have shown that infiltrating blood-derived macrophages are nonetheless crucial for neuroprotective and anti-inflammatory activities at the injury site; we have therefore proposed that the infiltrating cells fulfill specialized functions in the recovery process, which the resident immune cells either fail to display, or at least do not manifest at the right time or at sufficient levels (Shechter et al., 2009). In animal models of optic nerve injury, it was found that macrophages can modify the non-permissive nature of the optic nerve for regeneration *in vitro* (David et al., 1990), and that transplantation of activated macrophages into the injured optic nerve can facilitate regrowth *in vivo* (Lazarov-Spiegler et al., 1996). In line with these observations, the important contribution of a macrophage-derived molecule, oncomodulin, to the regeneration of the optic nerve, was identified by Benowitz and colleagues (Yin et al., 2006, 2009; Cui et al., 2009), who coined the term “inflammation-induced regeneration.” Collectively, these results attribute to innate immunity an important role in eye repair, and reveal the ability of macrophages to orchestrate neuroprotection and axonal regeneration.

The beneficial role of adaptive immunity in neuroprotection was initially observed in animal models simulating different aspects of glaucoma, where it was found that the extent of retinal ganglion cell loss is increased in immunocompromised animals relative to immunocompetent ones (Kipnis et al., 2001; Schori et al., 2001; Yoles et al., 2001; Bakalash et al., 2002). Moreover, T cell-based vaccinations, both passive and active, promote neuroprotection after optic nerve crush (Moalem et al., 1999; Fisher et al., 2001). Importantly, the potential benefit derived from T cells in these systems relies, at least in part, on a delicate balance between effector and regulatory subsets of these cells (Kipnis et al., 2002, 2004). More recently, results obtained in different models of CNS insult suggested that the beneficial effects of T cells might be mediated in part by controlling the recruitment of monocyte-derived macrophages from the circulation (Butovsky et al., 2007; Schwartz et al., 2009; Shechter et al., 2009). Thus, the well-orchestrated collaboration between the innate and adaptive arms of the immune system appears to be optimal for achieving neuroprotection.

The beneficial involvement of immune cells in the eye is also observed in diseases that are immune-induced, such as autoimmune posterior uveitis, a potentially blinding inflammatory condition affecting the retina and the choroid of the eye. Studies in experimental autoimmune uveitis (EAU), an animal model of human posterior uveitis, demonstrate the heterogeneity of immune cells along this disease. Beside the well-characterized pro-inflammatory cells known to initiate EAU, the uveitic eye is also endowed with regulatory immune populations (Robertson et al., 2002; Kerr et al., 2008; Caspi et al., 2011; London et al., under revision). These cells, including subsets of macrophages and T cells, act to limit inflammation, presumably bringing the disease to a state of equilibrium and remission.

**FIGURE 1:**
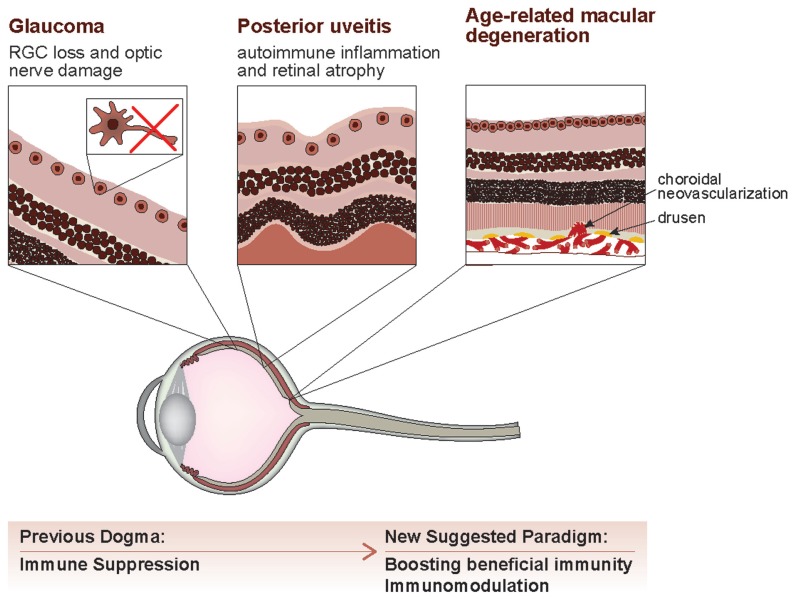
**An evolving view of immune involvement in the eye.** Ocular pathologies are initiated by multiple factors, and take on various manifestations. Glaucoma, a slowly progressing neurodegenerative disease, is characterized by loss of retinal ganglion cells and damage to the optic nerve. In posterior uveitis, retinal atrophy, and neuronal death are commonly induced by autoimmune inflammation, and age-related macular degeneration presents with drusen (“dry” AMD) and choroidal neovascularization (“wet” AMD). While the traditional dogma stated that immune privilege implies the exclusion of immune activity from the eye under any circumstances, our evolving understanding of immune privilege proposes that boosting beneficial immunity in the eye, in a well-regulated manner, rather than general immune suppression, is most favorable for coping with ocular pathologies, regardless of their initiating factors.

An additional pathology in which the immune system has been shown to fulfill various, perhaps opposing functions, is age-related macular degeneration (AMD), the leading cause of blindness in the elderly. Naturally, the etiology of AMD is very diverse; the disease is associated with numerous immune-related factors. Here too, the role of macrophages has been a matter of debate; on the one hand, it was found that aging is accompanied by a pathological shift to M2 macrophages, which are known to promote angiogenesis, and would therefore seem likely candidates for promoting choroidal neovascularization (CNV), the process by which abnormal blood vessels develop beneath the retina (Espinosa-Heidmann et al., 2003; Sakurai et al., 2003; Cao et al., 2011). On the other hand, studies have also shown that prevention of macrophage entry into the eye promotes CNV, whereas injection of macrophages inhibits it (Apte et al., 2006). Patel and Chan (2008) reviewed the seemingly contradictory functions of macrophages in AMD, and proposed that these conflicting findings reflect a dual role of macrophages in this pathology, where the uncontrolled pro-inflammatory M1 macrophages induce tissue damage, and the M2 macrophages, which are recruited to terminate the M1 response and to clear drusen and other age-related deposits, could also adversely affect disease progression by displaying pro-angiogenic activity. Among the additional factors associated with AMD pathogenesis and progression, a pivotal role has been attributed over the past several years to the complement system and its dysregulation (Klein et al., 2005; Patel and Chan, 2008; Anderson et al., 2010). These findings emphasize the need for a regulated immune response, in terms of timing, duration, and phenotype, and further support the argument that there are no “good” or “bad” immune cells; it is all a matter of their control and coordination. Moreover, the accumulating evidence on beneficial immune involvement in AMD and in the other ocular pathologies mentioned above give further reinforcement to the current contention that although the eye is an immune privileged site, it can enjoy the benefits of immune support, and thus immune regulation, rather than immune suppression, is the key to disease resolution, as in other parts of the body (**Figure 1**).

## A DIFFERENT VIEW OF IMMUNE PRIVILEGE

Immune privilege is an evolutionary adaptation aimed at protecting especially vulnerable organs from overwhelming inflammation that could abolish their functions and jeopardize the well-being of the individual. As vision is crucial for survival, it is understandable why the eye would be particularly protected from these risks (Streilein, 2003a). However, we propose that the immune privileged designation of the eye means that it has the privilege to enable selective immune responses most suitable and effective for its proper function in health and pathology. We contend that this is true for all other parts of the CNS, as well.

As many CNS pathologies are associated with local inflammation, they are generally treated with anti-inflammatory and immunosuppressive drugs. However, this treatment approach has shown limited success in animal models of ocular pathologies and other neurodegenerative disorders, as well as in the clinic, and in some cases was even found to exacerbate disease (Levin et al., 1999; Solberg et al., 1999; Bakalash et al., 2003; Ohlsson et al., 2004; Dimitriu et al., 2008; Schwartz and Shechter, 2010). The benefits of those drugs, if any, are often temporary, as they help relieve some of the symptoms but do not address the underlying pathological processes (Gronert, 2010). Bearing in mind the heterogeneity of immune cells and their changing functions along the course of disease, together with the delicate balance of counter-regulatory signals required for effective resolution of inflammation (Gronert, 2010), we suggest that a more efficient approach to treating such disorders would be to manipulate specific immune subsets in a timely manner, rather than to globally inhibit the immune response (**Figure 1**).

Finally, our interpretation of the privilege of immunity in immune privileged sites does not negate the possibility that under certain conditions, immune privilege is breached in order to preserve the life of the individual, at the expense of local loss of function; this is the case in certain microbial infections, or in the presence of highly immunogenic tumors (Morrison et al., 1989; Niederkorn, 1991; Li and Niederkorn, 1997; Streilein et al., 1997; Saint Andre et al., 2002; Niederkorn and Stein-Streilein, 2010), in which a powerful immune response is essential, and the risk of blindness is accepted for the sake of survival (Niederkorn and Stein-Streilein, 2010).

## Conflict of Interest Statement

The authors declare that the research was conducted in the absence of any commercial or financial relationships that could be construed as a potential conflict of interest.
